# The two-layered cross technique for post-over-the-scope clip bleeding

**DOI:** 10.1055/a-2678-9669

**Published:** 2025-08-27

**Authors:** Koichiro Kawano, Mamoru Takenaka, Koutaro Mine, Reiko Kawano, Daisuke Kagoshige, Katsuhisa Nishi, Masatoshi Kudo

**Affiliations:** 1 13865 Department of Gastroenterology, Hyogo Prefectural Awaji Medical Center, Sumoto, Japan; 2 Department of Gastroenterology and Hepatology, Kindai University Faculty of Medicine, Osakasayama, Japan

The over-the-scope (OTS) clip is a highly effective tool for hemostasis in gastrointestinal bleeding 1 2 3 4 ; however, bleeding may persist despite successful placement 1 5 . The reason is that a vessel that runs perpendicular to its closure may slip between the clip’s nail and hinge, resulting in failed hemostasis ( Fig. 1 ). When common hemoclips or ligature bands are used for post-OTS-clip bleeding, hemostasis is likely to be difficult due to interference of the implanted OTS clip or inadequate tissue aspiration. When a second OTS clip is used to try to stop bleeding, the occlusion of the second OTS clip tends to be in the same direction as the first OTS clip, and hemostasis may not be achieved ( Fig. 2 ).

**Fig. 1:**
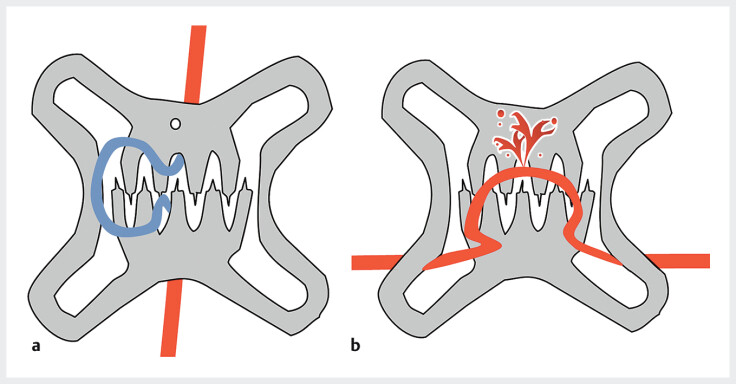
Schematic of initial over-the-scope (OTS) clip placement. **a** When the direction of occlusion of the OTS clip claw aligns with the direction of the vessel, the vessel is occluded by the claw, and hemostasis is achieved. **b** If the direction of occlusion of the OTS clip claw does not align with the vessel's direction, the blood vessel may pass between the clip's nail and hinge, resulting in failed hemostasis, even if the clip appears to be properly placed.

**Fig. 2:**
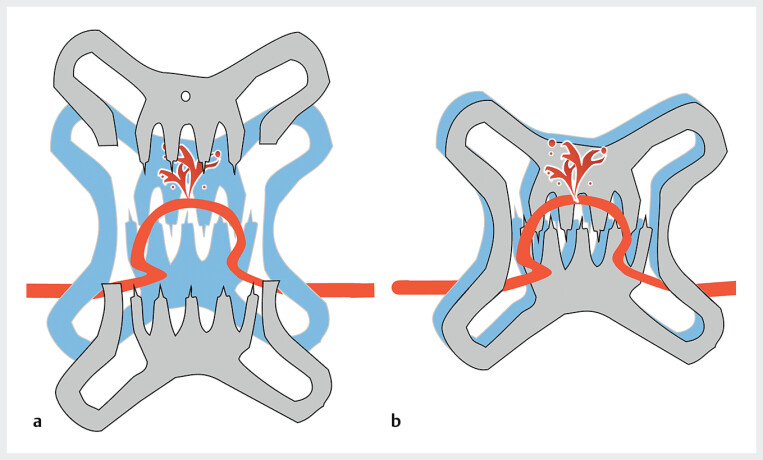
Schematic of a second OTS clip placed in the standard orientation for post-OTS-clip bleeding. **a** Before OTS clip release. **b** After OTS clip release. Since the orientation of the claws on both the first and second OTS clip is identical, the second clip may fail to stop the bleeding because the vessel may again pass between the nail and hinge.

To ensure hemostasis using a second OTS clip for post-OTS-clip bleeding, the occlusion direction of the second OTS clip should be orthogonal to that of the first OTS clip. However, rotating the endoscope in a tortuous gastrointestinal tract to change clip orientation may be technically challenging.

In general, the OTS clip’s orientation is determined by the position of the forceps hole at the endoscope tip. The clip is aligned by matching the traction thread with this hole to avoid obstructing the endoscopic view. The two-layered cross technique addresses this by rotating the second OTS clip 90° from its standard orientation ( Fig. 3 ), allowing orthogonal placement to the first OTS clip. This captures the vessel initially missed and ensures hemostasis ( Fig. 4 ).

**Fig. 3:**
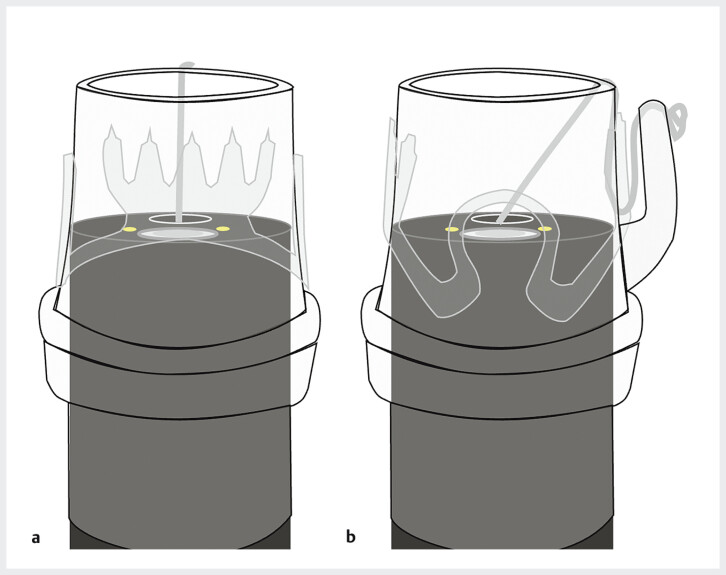
Schematic of endoscopic attachment of the OTS clip. **a** Under normal conditions, the forceps hole of the endoscope is aligned with the traction thread of the OTS clip. **b** In the two-layered cross technique, the OTS clip is rotated 90° from the standard position, causing the forceps hole and traction thread to become misaligned, with the traction thread entering the endoscopic field of view.

**Fig. 4:**
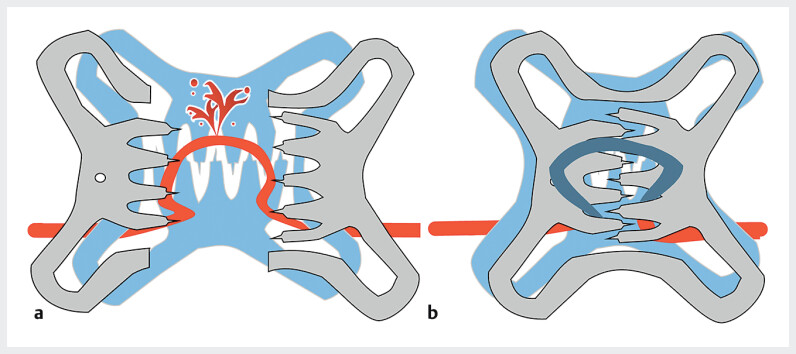
Schematic of a second OTS clip placed using the two-layered cross technique for post-OTS-clip hemorrhage. **a** Before OTS clip release. **b** After OTS clip release. The orientations of the claws of the first and second OTS clips are different. Therefore, the second OTS clip’s claw successfully occludes the vessel, allowing hemostasis.

In an 82-year-old woman with hematochezia and a visible bleeding vessel in the sigmoid diverticulum, hemostasis was initially achieved with an OTS clip. Bleeding recurred two hours later. A second OTS clip, applied orthogonally using the two-layered cross technique, achieved hemostasis ( Fig. 5 , Video 1 ). The patient was discharged without further bleeding after six days.

**Fig. 5:**
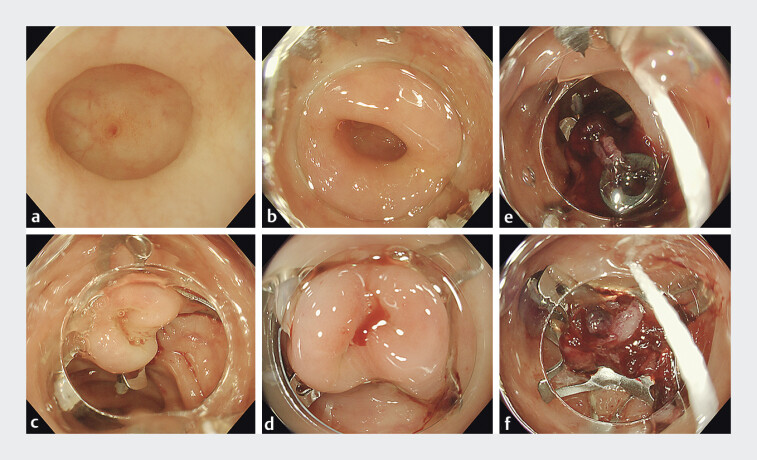
Endoscopic images of the hemostasis. **a** An exposed bleeding vessel is seen inside the sigmoid diverticulum. **b** Both claws of the OTS clip are positioned at the 4 and 10 oʼclock positions, and the OTS clipʼs traction thread is not visible in the endoscopic view. **c** The OTS clip is placed in the diverticulum, causing the inverted diverticulum to distend, and hemostasis is achieved. **d** The OTS clip remains in place, and bleeding is observed from the inverted and elevated diverticulum. **e** Repeated attempts at endoscopic band ligation of the inverted, elevated diverticulum fail to achieve hemostasis due to inadequate tissue grasping. A second OTS clip is placed, with both claws positioned at the 1 and 7 oʼclock positions, and the traction thread is visible in the endoscopic field of view. **f** The second OTS clip is placed over the superficial layer of the first clip using the two-layered cross technique. The claw orientation differs from the first placement, and hemostasis is achieved.

This video demonstrates the two-layered cross technique for post-OTS clip bleeding, enabling effective hemostasis in cases where initial clip placement is insufficient.Video 1

This technique may be an effective option for managing post-OTS-clip rebleeding.

Endoscopy_UCTN_Code_TTT_1AQ_2AG

## Notes

Endoscopy E-Videos https://eref.thieme.de/e-videos
*E-Videos* is an open access online section of the journal *Endoscopy* , reporting on interesting cases and new techniques in gastroenterological endoscopy. All papers include a high-quality video and are published with a Creative Commons CC-BY license. Endoscopy E-Videos qualify for HINARI discounts and waivers and eligibility is automatically checked during the submission process. We grant 100% waivers to articles whose corresponding authors are based in Group A countries and 50% waivers to those who are based in Group B countries as classified by Research4Life (see: https://www.research4life.org/access/eligibility/ ). This section has its own submission website at https://mc.manuscriptcentral.com/e-videos .
